# Late tumor pseudoprogression followed by complete remission after lung stereotactic ablative radiotherapy

**DOI:** 10.1186/1748-717X-8-167

**Published:** 2013-07-06

**Authors:** Michael C Stauder, Jessica W Rooney, Michelle A Neben-Wittich, Yolanda I Garces, Kenneth R Olivier

**Affiliations:** 1 Department of Radiation Oncology, MD Anderson Cancer Center, 1515 Holcombe Blvd., Unit 1202, Houston 77030, TX, USA; 2 Department of Radiation Oncology, Mayo Clinic, Rochester, MN, USA

**Keywords:** Radiotherapy, Stereotactic body radiotherapy, Thoracic neoplasms, Radiotherapy, Fluorodeoxyglucose F18

## Abstract

Lung stereotactic ablative radiotherapy (SABR) has recently become more common in the management of patients with early-stage non-small cell lung cancer (NSCLC) and metastatic lung lesions who are not surgical candidates. By design, SABR is applied to small treatment volumes, using fewer but significantly higher dose fractions, and steep dose gradients. This treatment theoretically maximizes tumor cell death and decreases the risk of damage to the surrounding normal tissues. Local control rates for SABR in early stage lung cancer remain high. Since the numbers of primary tumor recurrences is small, some debate exists as to the appropriate definition of treatment failure. Controversies remain regarding the most appropriate interpretation of imaging tests obtained to evaluate treatment outcomes after lung SABR. Most definitions of progression include an increasing diameter of target lesion which can be problematic given the known mass-like consolidation seen on CT imaging after ablative therapy. Here, we present a case report illustrative of the pitfalls of relying solely on anatomic imaging to determine SABR treatment failure.

## Background

The use of stereotactic ablative radiotherapy (SABR) has recently become more common in the management of patients with early-stage non-small cell lung cancer (NSCLC) and metastatic lung lesions who are not surgical candidates. Although the use of SABR continues to increase, controversies remain regarding the most appropriate interpretation of imaging tests obtained to evaluate treatment outcomes. Mass-like consolidation within the lung parenchyma after SABR can often mimic tumor progression making evaluation of patient outcomes difficult [1,2]. Likewise, the presence of FDG-avidity within the lung at the site of previous SABR can be due to lung inflammation or pneumonitis versus tumor progression [3]. Currently adopted standards to define treatment failure can often be contradictory in their use for defining disease progression [4-6]. The current case illustrates the need for a circumspect approach to imaging obtained after SABR and the utility of when to define tumor/disease progression.

## Case description

A 70-year old male presented to our clinic after a chest computed tomography (CT) scan revealed a speculated mass in the anteriomedial portion of the left upper lobe of the lung. A review of the patient’s imaging studies from 1 year prior shows the speculated mass to be a new finding. The patient’s past medical history consisted of severe cardiopulmonary disease, end stage emphysema, aortic stenosis, prior myocardial infarction, advanced peripheral vascular disease and history of coronary artery bypass graft. The patient is a current smoker and reported a 90 pack-year history of cigarette smoking. He required 3 liters/minute of oxygen by nasal cannula 24-hours per day. The patient was registered in a prospective, IRB-approved registry designed to collect pertinent clinical, pathologic and treatment data on those patients receiving SABR at Mayo Clinic.

**Figure 1:**
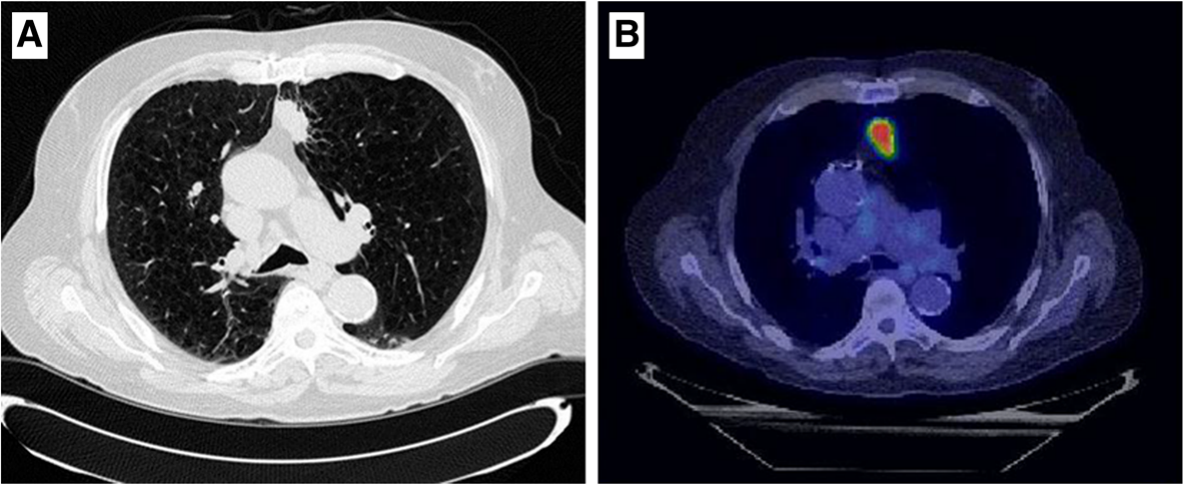
Chest CT and PET scan of a 70 year-old male revealed a 3.3 cm hypermetabolic mass with an SUV maximum of 12.3.

A fluorine-18 [^18^F] FDG Positron Emission Tomography (PET)/CT scan was performed for the purpose of staging and revealed a 3.3 cm hypermetabolic mass (SUV maximum 12.3) in the left upper lobe abutting the mediastinum (Figure 1). Radiologic report stated the nodule was worrisome for primary bronchogenic carcinoma. No evidence of FDG-avid metastatic disease was seen. Consultation by medical oncology, thoracic surgery and radiation oncology was obtained and due to his significant comorbidities, surgical resection was contraindicated. Likewise, due to poor pulmonary function, location of the lesion, and very high likelihood of malignancy, a biopsy of the FDG-avid lesion was not performed. Pulmonary function testing performed at diagnosis revealed a FEV1 of 0.74 (21% of predicted) with a DLCO of 39% predicted. Ultimately, the patient elected to be treated with SABR for definitive management of this FDG-avid presumed malignancy.

## Treatment

The patient received SABR to a total dose of 48 Gray in 4 consecutive daily fractions (Fx). Treatment planning was performed with full body immobilization and 4-dimensional CT-based planning and daily cone-beam CT (CBCT) for image guidance. Since January 2008, a total of 372 patients have been treated with lung SABR and our institutional technique has been described previously [7]. Briefly, each patient was immobilized using the Body-Fix whole-body immobilization system (Elekta, Stockholm, Sweden). Axial CT images were obtained on a General Electric Light Speed RT 16-slice CT simulator (GE Medical, Milwaukee, WI). Image acquisition was obtained at a 2.5-mm slice thickness. Four-dimensional CT imaging with respiratory monitoring was performed using Varian RPM (Palo Alto, CA) and an infrared reflector that was placed on the patient’s chest or upper abdomen. The patient’s respiratory pattern and CT data were linked by respiratory phase at the time of simulation. No abdominal compression was used. A treatment isocenter was established on the average CT data set on the GE Advantage virtual simulation station and normal tissue and tumor volume contouring was performed on the Advantage workstation. Fusion of the PET/CT data set from the patient’s staging study was used for assistance in tumor delineation. A gross tumor volume (GTV) of 7.9 cubic centimeters (cc) was defined using CT lung windows (1500, -500). An internal target volume (ITV) of 10.3 cc was created from the gross tumor volume, accounting for the movement of the tumor in three dimensions using the four-dimensional CT image data. The planning target volume (PTV) of 45 cc was created by a uniform 0.5 cm expansion of the ITV in the axially and 1.0 cm longitudinally. Normal tissue organs at risk, including the spinal cord, heart, skin, chest wall, heart, bronchial tree (including trachea), and esophagus were defined and dose constraints similar to TG 101 were used [8]. A total of 99.4% of the PTV received at least 48 Gy. Maximum PTV point dose was 6576 cGy (137%) and the mean dose to the PTV was 5645 cGy. The total lung received a mean dose of 350 cGy with mean dose to the left lung of 370 cGy and 326 Gy to theright lung. The conformality parameters for the treatment were 2917 cGy (61%), 4.23, and 1.13 for D_2cm_, R50 and R100, respectively. Prior to each treatment an initial CBCT scan was acquired, and a visual match performed. The prescribed treatment was delivered uneventfully and no acute treatment related side effects were observed.

## Follow-up

**Figure 2:**
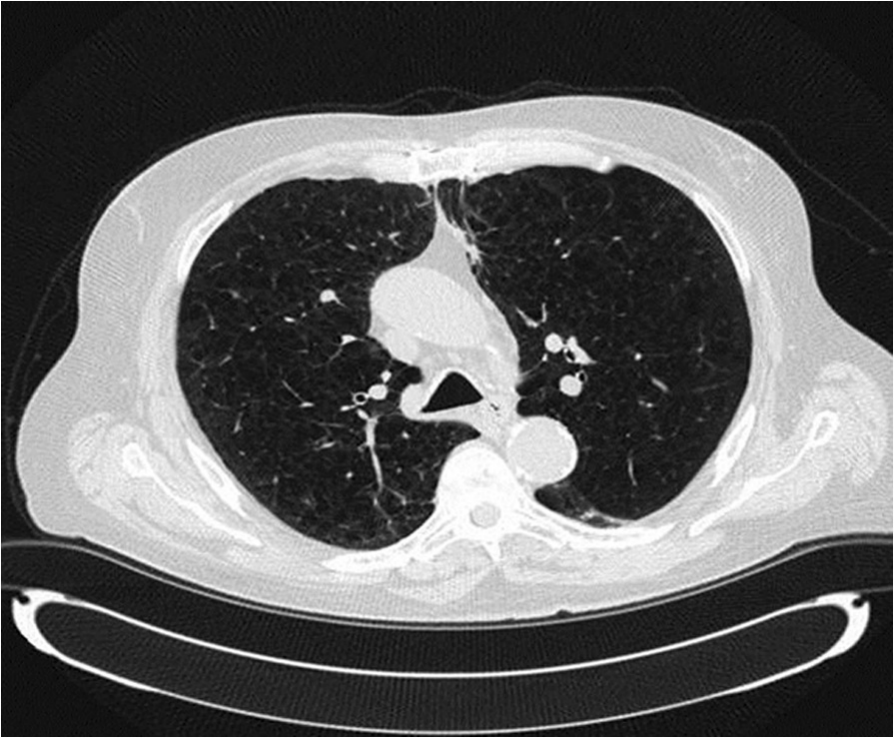
CT scan performed 2 months after SABR shows significant reduction in size of the treated lung lesion without evidence of radiation pneumonits.

The patient returned to clinic approximately 2 months after completing SABR and a CT scan of the chest (2.5 mm slice thickness) showed significant reduction in size of the treated lung lesion without radiologic or clinical evidence of radiation pneumonits (Figure 2). The patient reported no changes in his baseline dyspnea and continued on 3 liters oxygen by nasal cannula. At 12 months post-SABR, a CT scan of the chest revealed an increase in size of the target lesion to 9 mmwith 2–3 mm of growth since the scan obtained 6 months prior. Due to the small change in size the decision was made to continue close observation. Three additional CT scans obtained at 3 month intervals showed no further increase in the size of the lesion.

**Figure 3:**
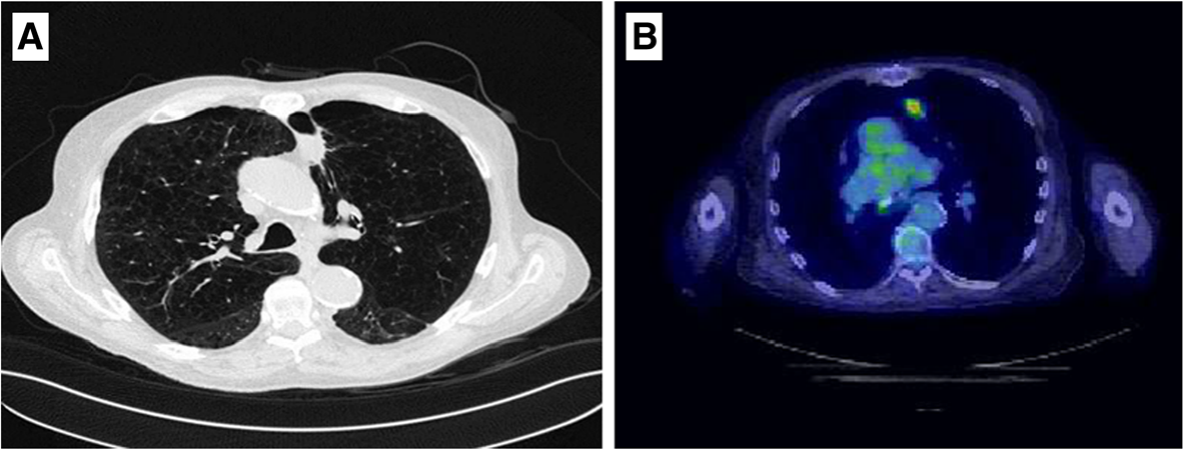
Follow-up CT scan at 30 months post-treatment revealed a 2.5 × 1.9 cm nodule in the previous treatment field with a SUVmax of 5.2.

**Figure 4:**
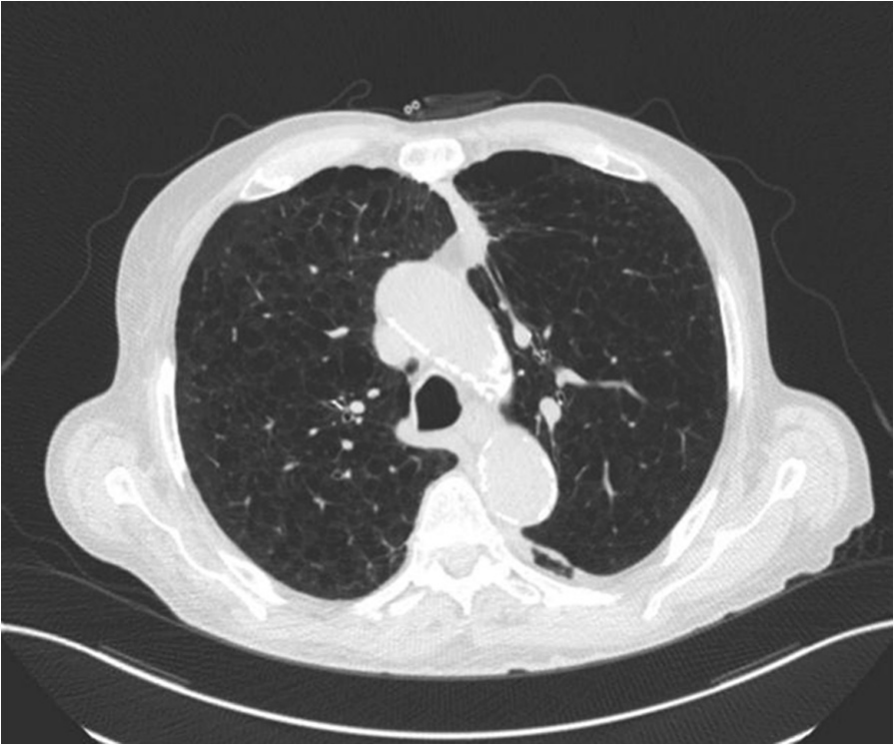
CT scan at 46-months of follow-up with residual consolidative mass at the site of the biopsied lesion.

30 months after treatment, a CT revealed a 2.5 × 1.9 cm nodule area within the previous treatment field. A PET scan was performed for further assessment and showed the nodule to be PET avid with a SUVmax of 5.2 (Figure 3). Also seen were new scattered small bilateral pulmonary nodules too small to evaluate with PET and a 9 mm anterior right LL nodule (not in the treatment field) which was not PET avid. The physicians involved in the patient’s care felt this new nodule represented local recurrence and the use of repeat SABR was entertained. As repeat SABR in an oxygen dependent patient was felt to be associated with significant risk the patient was counselled to undergo biopsy. A transbronchial biopsy was performed under the direction of pulmonary medicine. Pathologic review showed benign bronchial mucosa and bronchial wall with marked acute and chronic inflammation with bronchial washings showing scant cellularity but no evidence of malignancy. No further treatment was undertaken. Three months after the biopsy, the maximal SUV diminished to 2.8 and the associated soft tissue component had decreased in size measuring 1.5 cm. The previously identified 9 mm right lower lobe pulmonary nodule spontaneously resolved. There was no evidence of other pulmonary nodules or metastatic disease. With continued follow up, the biopsied nodule continued to decrease in size. The patient continues to do well at 46-months of follow-up, with complete resolution of the biopsied lesion on PET imaging (Figure 4). No evidence of new pulmonary lesions and no other changes in clinical status were noted. While some consolidative mass remains at 46 months, negative biopsy and the loss of FDG avidity within the lesion denote stable disease without evidence of recurrence.

## Discussion

The presented case report documents the apparent treatment failure by radiologic criteria of an early stage NSCLC after SABR. A negative biopsy of the target lesion and a total follow-up of 46 months indicate tumor pseudoprogression with no evidence of recurrent disease.

Local control rates after treatment with SABR for early stage lung cancer have been reported to be as high as 80-100% [9-13]. Since the numbers of primary tumor recurrences remain small, some debate exists as to the appropriate definition of treatment failure. Ongoing lung SABR clinical trials employ the RECIST criteria to assess target lesion response to treatment which defines progressive disease by two criteria 1) an increase in the total length of all measurable lesions of more than 20% (and at least 5 mm) compared with the smallest sum of lesion sizes or 2) the appearance of unequivocal new disease [4]. In addition to anatomical imaging such as CT and MRI, PET scan data can also be used in tumors with criteria meeting local enlargement (LE) status to help determine progression. Current RTOG trials state that a lesion should be avid on (PET)imaging with uptake of a similar intensity as the pre-treatment staging, but given the long interval history from treatment in our patient, this definition becomes problematic [14].

In an EORTC consensus statement, an increase in FDG tumor standard uptake value (SUV) of greater than 25% within the tumor region is termed progressive metabolic disease (PMD) [6], but no temporal relation is defined. Also, both the RECIST and EORTC definitions can be limited in the face of newer ablative radiation techniques such as SABR which may produce profound local inflammatory responses. As a result, attempts to better define the PET criteria of treatment failure have recently been initiated. In the recently described PERCIST 1.0 model, metabolic progression is defined as a 30% increase in theSUV corrected for lean body mass, new [^18^F]FDG–avid lesions, or growth in lesion total glycolysisby more than 75% [5]. The lesion described in this case report, by either RECIST 1.1 or PERCIST 1.0 criteria, would be categorized as a partial response (PR) in the post therapy setting and subsequently as progressive disease (PD) by RECIST or as progressive metabolic disease (PMD).

In general, the SUV_max_ measured on post-SABR PET imaging decreases in a linear fashion. This is shown in one recent study analyzing the SUV before and after SABR in patients with early stage NSCLC [15]. Prior to SABR, the 14 patients evaluated in this pilot study had a median SUV of 8.7 with 6 of 13 maintaining a persistent SUV_max_> 3.5 at 12 months post-treatment. Recently, authors from MD Anderson Cancer Center reported on the use of PET to assess local failure after lung SABR [3]. In the 128 patients evaluated, a cut-off of SUV max of 5.0 was found to have 100% sensitivity and 91% specificity for tumor recurrence. Additionally, the negative predictive value if obtained at least 6 months after treatment was 100%.

Our patient is unique in that near complete response was seen on post treatment CT scans and all PET scans within the first 30 months after treatment were unremarkable for any FDG-avid lesions suggesting progression of disease. Another recent study of 80 patients receiving SABR for early-stage peripheral NSCLC reports that the median latency time for developing a mass-like consolidation in the area of treatment was 7 and 12 months in patients with and without tumor recurrence, respectively. Additionally, in all patients with tumor recurrence, consecutive rises in the volume of mass-like consolidation on serial imaging at 3-month intervals was seen [16]. The development of consolidation seen on CT scan may occur in 68-74% of patients receiving lung SABR, but only 11-30% truly represent recurrent tumor [1,2].

Given the typically short timeframe to recurrence seen after lung SABR as well as the high incidence of mass-like consolidation seen on follow-up imaging, one could potentially propose a new definition for tumor progression. Since tumor progression after SABR is an uncommon phenomenon and salvage treatment options are usually limited and associated with progressive risk, patience is warranted. Using the cut-off of three consecutive rises at 3-month intervals in the volume of mass-like consolidation as well as a two PET scans showing a SUV_max_ of ≥ 5.0 both obtained at least 6 months after completion of SABR is a reasonable definition for tumor progression. Either of these findings should warrant tissue confirmation with the caveat that the sensitivity of biopsy is not known for certain. One patient in a recent case series of surgical salvage following SABR, for example required 11 biopsy attempts for pathologic confirmation of recurrence [17].

Based on a recently published systemic review, a proposed algorithm for imaging follow up of SABR patients who are candidates for salvage treatment can be utilized [18]. The patient presented here, if classified according to that algorithm would have received the same anatomic and functional imaging. Therefore, the algorithm may serve as a framework to evaluate the optimal definitions of recurrence after SABR. A patient cohort with a greater number of treatment failures would be needed to test this definition prior to being put into clinical practice, but in the current case, a potentially harmful biopsy in a patient with limited underlying pulmonary function may have been avoided.

## Conclusion

Current definitions of tumor recurrence after lung SABR may overestimate the true incidence of progression. The case presented here highlights the need to develop a definition which incorporates anatomic, functional and kinetic information to increase the diagnostic sensitivity and specificity of tumor recurrence after lung SABR.

## Consent

Written informed consent was obtained from the patient for publication of this case report and any accompanying images.

## Competing interests

All authors declare that they have no competing interests.

## Authors’ contributions

MCS drafted the manuscript and conceived the report. JWR participated in image acquisition and manuscript editing. MNW is the responsible clinic physician and edited the manuscript. YIG is responsible for the technical treatment design and follow-up recommendations on an institutional/ departmental level. KRO participated in coordination of clinical studies and drafting of the manuscript. All authors have read and approved the final manuscript.
